# G_o _contributes to olfactory reception in *Drosophila melanogaster*

**DOI:** 10.1186/1472-6793-9-22

**Published:** 2009-11-28

**Authors:** Abhishek Chatterjee, Gregg Roman, Paul E Hardin

**Affiliations:** 1 Department of Biology and Center for Biological Clock Research, Texas A&M University, College Station, TX 77843, USA; 2 Department of Biology and Biochemistry, University of Houston, Houston, TX 77204, USA

## Abstract

**Background:**

Seven-transmembrane receptors typically mediate olfactory signal transduction by coupling to G-proteins. Although insect odorant receptors have seven transmembrane domains like G-protein coupled receptors, they have an inverted membrane topology and function as ligand-gated cation channels. Consequently, the involvement of cyclic nucleotides and G proteins in insect odor reception is controversial. Since the heterotrimeric G_o_α subunit is expressed in *Drosophila *olfactory receptor neurons, we reasoned that G_o _acts together with insect odorant receptor cation channels to mediate odor-induced physiological responses.

**Results:**

To test whether G_o _dependent signaling is involved in mediating olfactory responses in *Drosophila*, we analyzed electroantennogram and single-sensillum recording from flies that conditionally express pertussis toxin, a specific inhibitor of G_o _in *Drosophila*. Pertussis toxin expression in olfactory receptor neurons reversibly reduced the amplitude and hastened the termination of electroantennogram responses induced by ethyl acetate. The frequency of odor-induced spike firing from individual sensory neurons was also reduced by pertussis toxin. These results demonstrate that G_o _signaling is involved in increasing sensitivity of olfactory physiology in *Drosophila*. The effect of pertussis toxin was independent of odorant identity and intensity, indicating a generalized involvement of G_o _in olfactory reception.

**Conclusion:**

These results demonstrate that G_o _is required for maximal physiological responses to multiple odorants in *Drosophila*, and suggest that OR channel function and G-protein signaling are required for optimal physiological responses to odors.

## Background

Most animals rely on olfaction for foraging, predator and toxin avoidance, and social interactions. Odorants are detected by 7-transmembrane receptors, which normally transduce olfactory signaling by activating G-proteins. However, recent work in the fruit fly *Drosophila melanogaster *demonstrates that insect odorant receptors (ORs) act as ligand gated [1,2] and cyclic nucleotide gated [2] cation channels, and thus do not function as traditional G-protein coupled receptors. The Gα protein(s) responsible for inducing the production of cyclic nucleotides that activate cation channels formed by OR-complexes have not been identified, although G_q _has been implicated in *Drosophila *olfactory transduction [3]. Another Gα protein, G_o_, is expressed in the odorant receptor neurons (ORNs) of antenna from *Drosophila*, the silk moth *Bombyx mori*, and the mosquito *Anopheles gambae*, suggesting the functional involvement of G_o _in insect olfaction [4-7]. Although definitive immunohistochemical proof for dendritic localization of G_o _in olfactory sensilla is lacking, previous studies could not rule out the possibility of G_o _expression in ORN dendrites.

In *Drosophila*, the S1 subunit of pertussis toxin (PTX) selectively ADP-ribosylates G_o_, thereby inhibiting G_o _signaling [8,9]. We have employed existing and newly developed tools for controlling the spatial and temporal expression of PTX to investigate how G_o _inactivation affects physiological responses to odorants [10,11]. Loss of G_o _signaling in ORNs reduced the amplitude and enhanced the termination of EAG responses, and decreased odor-induced spike frequency in individual ORNs independent of odor type or concentration. These results demonstrate that G_o _is involved in modulating olfactory responses in *Drosophila*.

## Results and Discussion

**Figure 1:**
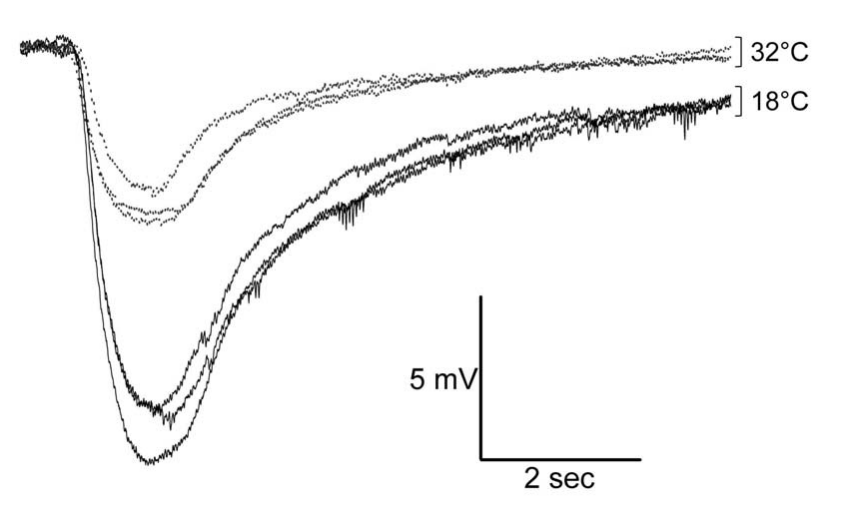
**PTX reduces the amplitude of ethyl acetate induced EAG responses**. EAG traces evoked by the application of 10^-4 ^ethyl acetate in Gal80^ts20^/*Or83b*-Gal4; UAS-PTX/+ flies at temperatures that restrict (18°C, black lines) or permit (32°C, gray lines) PTX expression.

**Figure 2:**
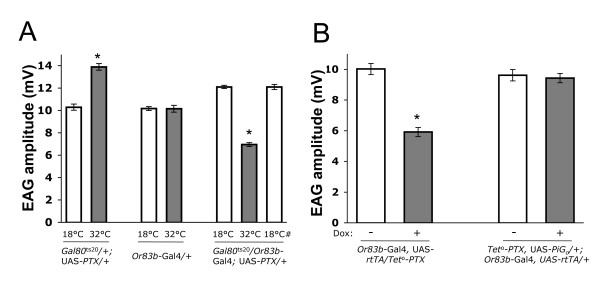
**Inhibition of heterotrimeric G_o _signaling reversibly reduces the amplitude of ethyl acetate evoked EAG responses**. For all fly strains, a 10^-4 ^dilution of ethyl acetate was used to evoke EAG responses. (A) EAG responses from *Gal80*^ts20^/+; UAS-PTX/+ and *Or83b*-Gal4/+ control strains do not decrease (p > 0.5) at 32°C compared to 18°C. *Gal80*^ts20^/*Or83b*-Gal4; UAS-PTX/+ flies have a significantly (p < 0.0001) higher EAG amplitude in the absence of PTX expression before heat induction (18°C) or after recovery from heat induction (18°C#) than in the presence of PTX expression (32°C). (B) EAG responses from *Or83b*-Gal4; UAS-*rtTA*/*Tet*^o^-PTX flies are significantly (p < 0.0001) higher in the absence of PTX expression (dox -) than in the presence of PTX expression (dox +). EAG responses from flies that express PTX-insensitive G_o _(PiG_o_) in ORNs (*Tet*^o^-PTX, UAS-PiG_o_/+; *Or83b*-Gal4, UAS-*rtTA*) are not different (p > 0.7) whether PTX expression in ORNs is induced (dox +) or uninduced (dox -). For each genotype and treatment, at least 12 EAG recordings from minimum 6 flies were analyzed. Asterisks denote a significant (p < 0.05) change. All values are mean ± S.E.M.

To determine whether G_o _signaling mediates olfactory responses, EAG measurements were carried out on flies in which the widespread olfactory receptor neuron driver *Or83b*-Gal4 was used to drive UAS-PTX in ORNs [12]. Conditional expression of PTX was achieved using the *Gal80*^ts20 ^TARGET system; at 18°C, functional *GAL80*^ts20 ^binds to and inhibits GAL4 and at 32°C GAL80^ts20 ^is inactivated thus allowing PTX expression [13]. At 32°C, *Or83b *promoter driven GAL4 was free to drive the transcription of PTX and inactivate *Drosophila *G_o _[10]. As a result, Gal80^ts20^/Or83b-Gal4; UAS-PTX/+ flies, which show a ~12 mV EAG amplitude to 10^-4 ^ethyl acetate at 18°C, produce a significantly (p < 0.0001) decreased EAG amplitude of ~7 mV at 32°C (Fig 1, Fig 2A). This result demonstrates that PTX-sensitive G_o _is needed for high amplitude EAG responses, suggesting that G_o _is involved in generating receptor potential. To insure that the observed decrease in EAG amplitude did not arise from cell damage and/or cell death, we placed temperature-treated flies to 18°C for 24 hours and measured EAG responses. These flies regained normal EAG amplitude of ~12 mV, demonstrating that the effect of PTX is reversible (Fig 2A). Moreover, EAG responses evoked by 10^-4 ^ethyl acetate in *Gal80*^ts20^/+; UAS-PTX/+ and *Or83b*-Gal4/+ control strains did not show a decreased (p > 0.5) amplitude when the temperature was increased from 18°C to 32°C (Fig 2A), thus decreased amplitude does not result from an increased temperature. Temperature did induce a moderate increase in EAG amplitude in *Gal80*^ts20^/+; UAS-PTX/+ control flies, but this is likely due to the *Gal80*^ts20 ^transgene genetic background since *Gal80*^ts20^/+ flies displayed a modest increase in EAG amplitude when temperature was increased to 32°C (Additional file 1).

To confirm that PTX suppressed EAG amplitude, PTX was conditionally expressed in ORNs by combining the Gal4/UAS and tetracycline (Tet)-inducible Tet-On transactivator (Tet-On TA) systems [14]. Or83b-Gal4 was used to drive expression of UAS-rtTA (reverse tetracycline transactivator) in ORNs. In the presence of the tetracycline analog doxycycline, rtTA binds to the tet-operator (tet^o^) and activates transcription of the tet^o^-PTX transgene. Upon addition of doxycycline, PTX expression suppressed (p < 0.0001) EAG amplitude by ~40% (Fig 2B). To insure that PTX suppressed EAG amplitude by inhibiting G_o_, a PTX insensitive G_o_α (PiG_o_) was expressed along with PTX in ORNs. Doxycycline-induced PTX expression did not affect (p > 0.7) EAG amplitude in flies expressing PiG_o _in ORNs, demonstrating that PiG_o _completely rescued the action of PTX on endogenous G_o _(Fig 2B). These results map the effects of PTX to G_o _and confirm that G_o _signaling contributes to olfactory responses.

**Figure 3:**
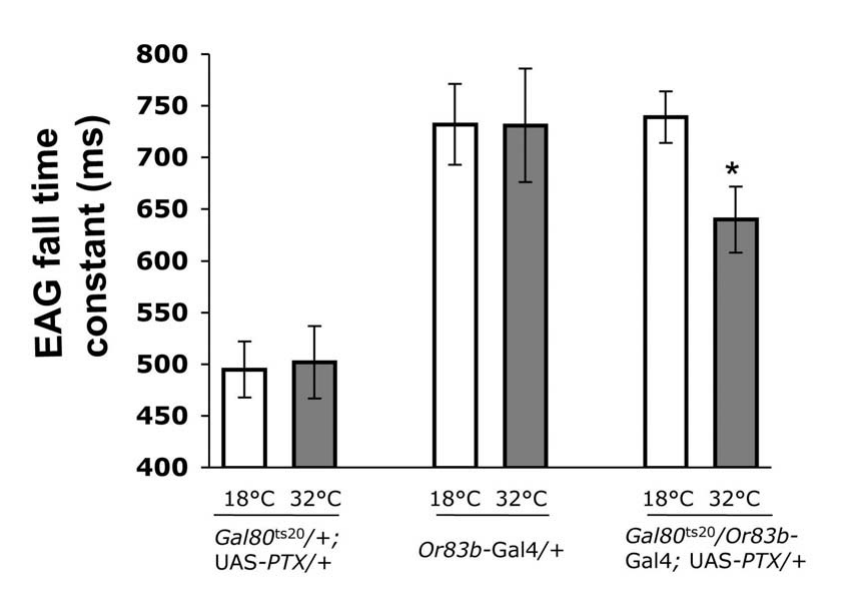
**G_o _activity is required for the perdurance of EAG responses**. For each fly strain, a 10^-4 ^dilution of ethyl acetate was used to evoke EAG responses. The EAG fall time constant in *Gal80*^ts20^/+; UAS-PTX/+ and *Or83b*-Gal4/+ control strains is not different (p > 0.8) at 18°C and 32°C. *Gal80*^ts20^/*Or83b*-Gal4; UAS-PTX/+ flies have a significantly (p < 0.01) longer EAG fall time constant in the absence of PTX expression (18°C) than in the presence of PTX expression (32°C). For each genotype and temperature, at least 8 EAG recordings from minimum 4 flies were analyzed. Asterisks denote a significant (p < 0.05) change. All values are mean ± S.E.M.

To investigate the effect of PTX on EAG dynamics, we looked at fall time constant (τ_f_) as a measure of the termination kinetics of EAG responses. Fall time constant is the time necessary to recover one-third of the maximal EAG amplitude after stimulation. This parameter is independent of amplitude, and unlike amplitude τ_f _remains relatively unaffected by small changes in electrode placement [15]. Upon stimulation for 500 ms with 10^-4 ^ethyl acetate, τ_f _was significantly (p < 0.01) lowered in the *Gal80*^ts20^/*Or83b*-Gal4; UAS-PTX/+ flies at 32°C compared to that at 18°C, whereas the two control strains showed no effect (p > 0.8) of temperature on τ_f _(Fig 3). For a given odorant, τ_f _decreases if either the concentration of the odorant or its delivery duration is reduced [15]. Inhibition of G_o _resulted in faster termination kinetics typically seen in control flies upon application of a 10-fold lower dose of odorant (Additional file 2). Since inactivation of G_o _shortened τ_f_, it can be argued that transduction of odor-information in the antenna was impaired in absence of G_o_. Our observation that G_o _is needed for the persistence of the electrophysiological response *in vivo *corroborates the *in vitro *results that implicate G-protein mediated signal amplification in prolonged odor signaling [2].

**Figure 4:**
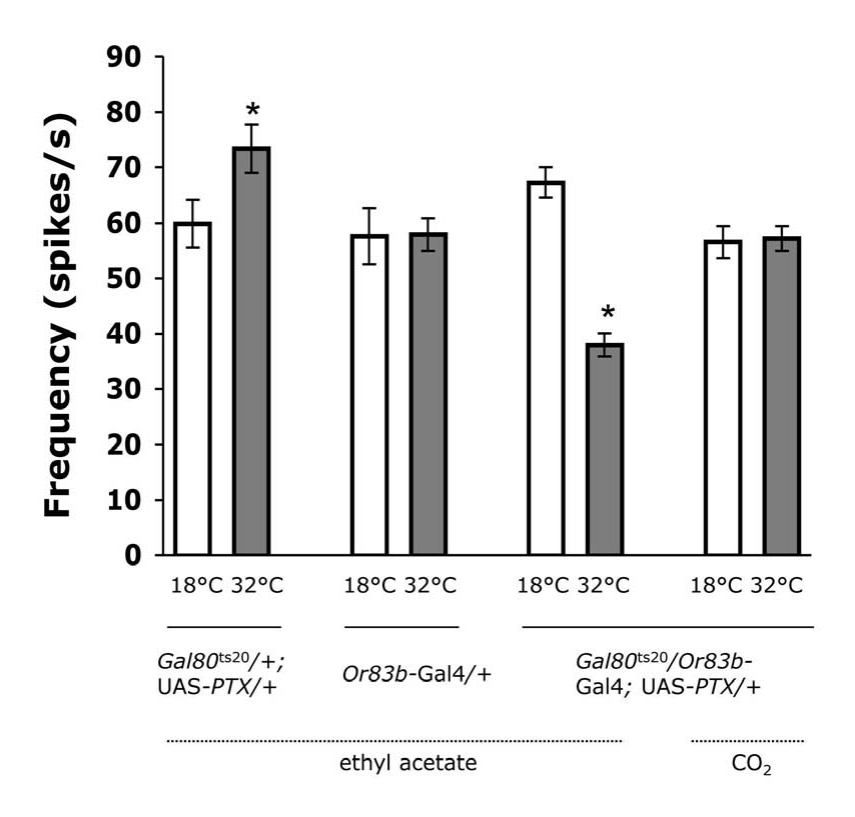
**G_o _inhibition reduces odor-evoked firing frequency**. For each fly strain, CO_2 _and a 10^-4 ^dilution of ethyl acetate were used to evoke spike activity from ab1C or ab1A neurons respectively. Spike frequency in *Gal80*^ts20^/+; UAS-PTX/+ and *Or83b*-Gal4/+ control strains do not decrease (p > 0.5) at 32°C compared to 18°C. *Gal80*^ts20^/*Or83b*-Gal4; UAS-PTX/+ flies have a significantly (p < 0.0001) lower ethyl acetate evoked ab1A spike frequency in the absence of PTX expression (18°C) than in the presence of PTX expression (32°C), whereas CO_2_-induced single unit responses in the ab1C neuron was not unaffected (p > 0.8). For each genotype and temperature, responses from at least 8 ORNs from minimum 4 flies were analyzed. Asterisks denote a significant (p < 0.05) change. All values are mean ± S.E.M.

Odor-induced EAG responses are thought to mainly consist of the summation of receptor potentials of many ORNs in close proximity to the recording electrode [16]. However, it is difficult to correlate EAG responses with single cellular processes that occur when individual ORNs respond to odorants. The limited resolution of EAGs can be overcome by recording single unit responses from individual sensilla. In contrast to EAG responses, single unit recordings consist of spikes that represent extracellularly recorded action potentials of individual ORNs in the sensillum [17]. To investigate the role of G_o _at the level of single cell physiology, we performed single-sensillum recording on ab1 sensilla whose 'A' neuron (i.e. the neuron producing the largest 'A' spike) is known to robustly respond to ethyl acetate [18]. Expression of PTX significantly (p < 0.0001) reduced the ethyl acetate-evoked firing frequency of ab1A spikes (Fig 4). However, the spontaneous firing frequency did not (p > 0.2) change, indicating that inactivation of G_o _did not alter the physiology of uninduced resting membrane (Additional file 3). The same sensillum houses the CO_2_-sensing ab1C neuron [18-20], which does not express *Or83b*-driven PTX. CO_2_-induced single unit responses are not affected by *Or83b*-driven PTX in ab1C neurons (Fig 4), thus confirming the specificity of our gene expression system. The reduction in ethyl acetate induced spike frequency was not a mere physical response caused by increase in temperature because the two control strains did not show any decrease (p > 0.5) in firing frequency in response to increased temperature. Inhibition of G_o _signaling lowered the odor-induced frequency of ab1A spikes and odor-evoked EAG response by an equivalent amount, *i.e*., 40-45% reduction in response. Taken together, these results reveal that G_o _plays an important role in olfactory reception within the *Drosophila *ORNs.

**Figure 5:**
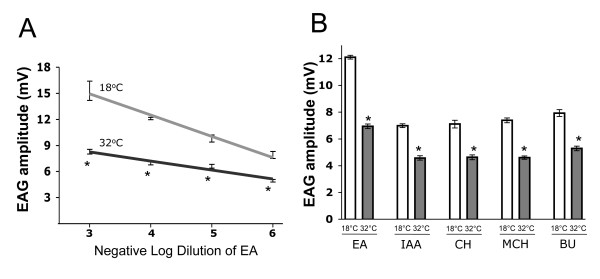
**G_o _signaling is required for normal EAG responses to diverse odorants**. (A) EAG responses evoked by four different concentrations of ethyl acetate (EA) in *Gal80*^ts20^/*Or83b-Gal4*; UAS-PTX/+ flies are significantly (p < 0.0001) higher in the absence of PTX expression (18°C) than in the presence of PTX expression (32°C). (B) EAG responses evoked by a 10^-4 ^concentration of ethyl acetate (EA), a 10^-4 ^concentration of isoamyl acetate (IAA), a 10^-4 ^concentration of cyclohexanone (CH), a 10^-4 ^concentration of 4-methylcylcohexanol (MCH), and a 10^-3 ^concentration n-butanol (BUT) in *Gal80*^ts20^/*Or83b-Gal4*; UAS-PTX/+ flies are significantly (p < 0.0001) higher in the absence of PTX expression (18°C) than in the presence of PTX expression (32°C). For a given genotype, odor concentration, and temperature, at least 8 EAG recordings from minimum 4 flies were analyzed. Asterisks denote a significant (p < 0.05) change. All values are mean ± S.E.M.

To determine whether inhibition of G_o _signaling impairs olfactory responses only at certain concentrations of ethyl acetate, we recorded EAG responses in both PTX expressing and PTX non-expressing *Gal80*^ts20^/*Or83b*-Gal4; UAS-PTX/+ flies exposed to various concentrations of ethyl acetate (Fig 5A). PTX was found to repress EAG responses over a 1000-fold range of stimulus intensities (p < 0.0001); although the degree of repression was slightly higher at high concentrations of ethyl acetate. This effect was in contrast with the odor-intensity dependent effect of dG_q3_RNAi in behavioral response of *Drosophila *to odors [21]. Odor sensitivity was compared by noting the increase in odor concentration that is needed in G_o_-compromised flies to elicit EAG responses as high as that in flies with unaffected G_o_(see Materials and Methods). Comparison of the two dose-response curves reveals that PTX mediated suppression of EAG response is associated with a ~470 fold difference in sensitivity to ethyl acetate (Fig 5A).

We next determined whether G_o _contributes to the detection of odorants by other classes of sensilla. We chose a small panel of odorants, which included two acetates (ethyl acetate, isoamyl acetate) perceived by basiconic sensilla, one ketone (cyclohexanone) known to activate a single class of coeloconic sensilla, an alcohol (4-methyl-cylcohexanol) that is detected by trichoid and coeloconic sensilla, and another alcohol (n-butanol) that is detected by basiconic and coeloconic sensilla [18,22-24]. Our odor panel contained both attractants (e.g. ethyl acetate at 10^-4 ^concentration) and repellents (e.g. 4-methyl-cyclohexanol at 10^-4 ^concentration). EAG recordings revealed that PTX expression significantly (p < 0.0001) repressed EAG amplitudes to all five odorants tested (Fig 5B). In each case, the EAG amplitude was reduced by 38 ± (+/- 5) percent. These results suggest that G_o _plays a role in olfactory signaling across multiple classes of sensilla independent of odor identity or concentration.

Our results show that sensory signals from five odorants, including ethyl acetate, are modulated via G_o _signaling. These findings support the possibility that a single odorant may activate multiple transduction pathways since previous studies showed that G_q _is needed for optimal responses to isoamyl acetate, ethyl acetate and butanol [3,21]. Activation of *Drosophila *OR cation channel function by multiple odorants implies that both OR channel function and G-protein signaling are required for optimal responses to a given odor [1]. It is possible that odor bound ORs directly activate G_o _and G_q_, thus reinforcing and optimizing the ORN response by modulating second messenger levels.

## Conclusion

Our results demonstrate that G_o _is required for maximal physiological responses to a diverse group of attractive and aversive odorants in *Drosophila*. Given that diminished physiological responses to odors persist in the absence of G_o _signaling, it is likely that OR channel function, along with G-protein signaling, are required for optimal physiological responses to odors.

## Methods

### Generation of transgenic flies

The *Gal80*^ts20^; UAS-PTX.16 and UAS-rtTA901 were both previously described [10,14]. The Pertussis insensitive G_o_α was generated through the incorporation of the cysteine_351 _to isoleucine mutation in the wild type GM1620 G_o_α 47A cDNA by PCR. This pertussis insensitive G_o_α cDNA (PiG_o_) was cloned into the pPUAST vector [25]. The tet^0^-Pertussis toxin construct was assembled in two parts. The PTX coding region was cloned by PCR from pPUAS-PTX to include a *Pst*I site at the 5' end and an *Eco*RI site at the 3' end [10]. This construct was directly cloned into the *Pst*I and *Eco*RI sites of the pUSC1.0 vector [26]. The SV40 polyadenylation sequence from pBRETU was subsequently cloned as an *Eco*RI fragment behind the PTX coding sequences to generate the pPtet^o^-PTX vector [27]. Transgenic PUAS-PiG_o _and Ptet^o^-PTX flies were generated through direct embryo injection by Rainbow Transgenetic Flies, Inc. (Newbury Park, CA).

### Electrophysiological recording techniques

EAG and single-sensillum recording experiments were performed as previously described [28,29]. Recordings were carried out during the middle of the day on 2-5 day old flies raised at 18°C. Temperature sensitive *GAL80*^ts20 ^was inactivated by placing flies at 32°C for 18 hours. Heat-treated flies were then kept at 18°C for 24-48 hours for recovery. The Tet-On system was activated by feeding flies a 2% sucrose solution containing 2 mM doxycycline overnight. Dilutions of all odorants except CO_2 _were made in mineral oil. Odors were delivered for approximately 500 ms. At least eight EAG or single unit recordings from at least four different flies were analyzed for each data point. To quantify spike frequency, recordings from 10 different ORNs from at least 4 different flies were analyzed. Spikes were manually sorted and spontaneous frequency was not subtracted from the odor-induced net response. Statistical significance with respect to pairwise comparison was calculated using Student's t-test, and multiple means were compared by one-way ANOVA. The Bonferroni test was used for post hoc analyses. The PTX-induced change in sensitivity to ethyl acetate was calculated using a fitted linear equation (EAG amplitude = -2.45 × negative log dilution of ethyl acetate + 22.3) derived from the dose response curve from PTX non-expressing flies. A response of 8.4 mV to a 10^-3 ^dilution of ethyl acetate in PTX expressing flies equates to a 10^-5.67 ^dilution of ethyl acetate in PTX non-expression flies, or a ~470-fold reduction in stimulus concentration to produce the same response.

## Abbreviations

ORNs: Olfactory receptor neurons; ORs: odorant receptors; EAG: electroantennogram; EA: ethyl acetate; PTX: pertussis toxin; G_o_: heterotrimeric G-protein (o); tet: tetracycline; dox: doxycycline; PiG_o_: PTX insensitive G_o_α; τ_f_: fall time constant; tet^o^: tet-operator.

## Authors' contributions

AC conceived the study, participated in experimental design and the interpretation of results, carried out all electrophysiology experiments, and drafted the manuscript. GR participated in experimental design and the interpretation of results, constructed the PUAS-PiG_o _and Ptet^o^-PTX plasmids used to generate transgenic flies, and helped to edit the manuscript. PEH participated in experimental design and the interpretation of results, coordinated experiments, and edited the manuscript. All authors read and approved the final manuscript.

## Supplementary Material

Additional file 1 **Genetic background of *Gal80*^ts20 ^transgene causes a temperature-dependent increase in EAG amplitude**. EAG responses to ethyl acetate in wild-type controls are not different (p > 0.65) at 18°C and 32°C. EAG responses to ethyl acetate in *Gal80*^ts20^/+flies are significantly (p < 0.005) higher at 32°C than 18°C. For each genotype and temperature, at least 8 EAG recordings from minimum 4 flies were analyzed. Asterisks denote a significant (p < 0.05) change. All values are mean ± S.E.M. Click here for file

Additional file 2 **EAG Fall Time Constant is a function of odor intensity**. The decay of EAG response in *Gal80*^ts20^/*Or83b*-Gal4; UAS-PTX/+ flies at 18°C becomes faster as the concentration of ethyl acetate (EA) decreases (open circles). The EAG τ_f _in response to a 10^-4 ^dilution of ethyl acetate in *Gal80*^ts20^/*Or83b*-Gal4; UAS-PTX/+ flies at 32°C (closed circle) is equivalent to that of *Gal80*^ts20^/*Or83b*-Gal4; UAS-PTX/+ flies at 18°C in response to a 10^-5 ^dilution of ethyl acetate. For every dilution of ethyl acetate, at least 8 EAG recordings from minimum 4 flies were analyzed. All values are mean ± S.E.M. Click here for file

Additional file 3 **G_o _inactivation does not alter spontaneous firing frequency**. The frequency of spontaneous spikes in *Gal80*^ts20^/*Or83b*-Gal4; UAS-PTX/+ flies is not different (p > 0.21) when G_o _signaling is intact (18°C) or blocked by PTX expression (32°C). For each genotype and temperature, responses from at least 8 ORNs from minimum 4 flies were analyzed. All values are mean ± S.E.M. Click here for file
