# Supplementary figures and images for: Go contributes to olfactory reception in Drosophila melanogaster
Source: BMC Physiol. 2009 Nov 28;9:22. doi: 10.1186/1472-6793-9-22 (PMC2789035; doi:10.1186/1472-6793-9-22)

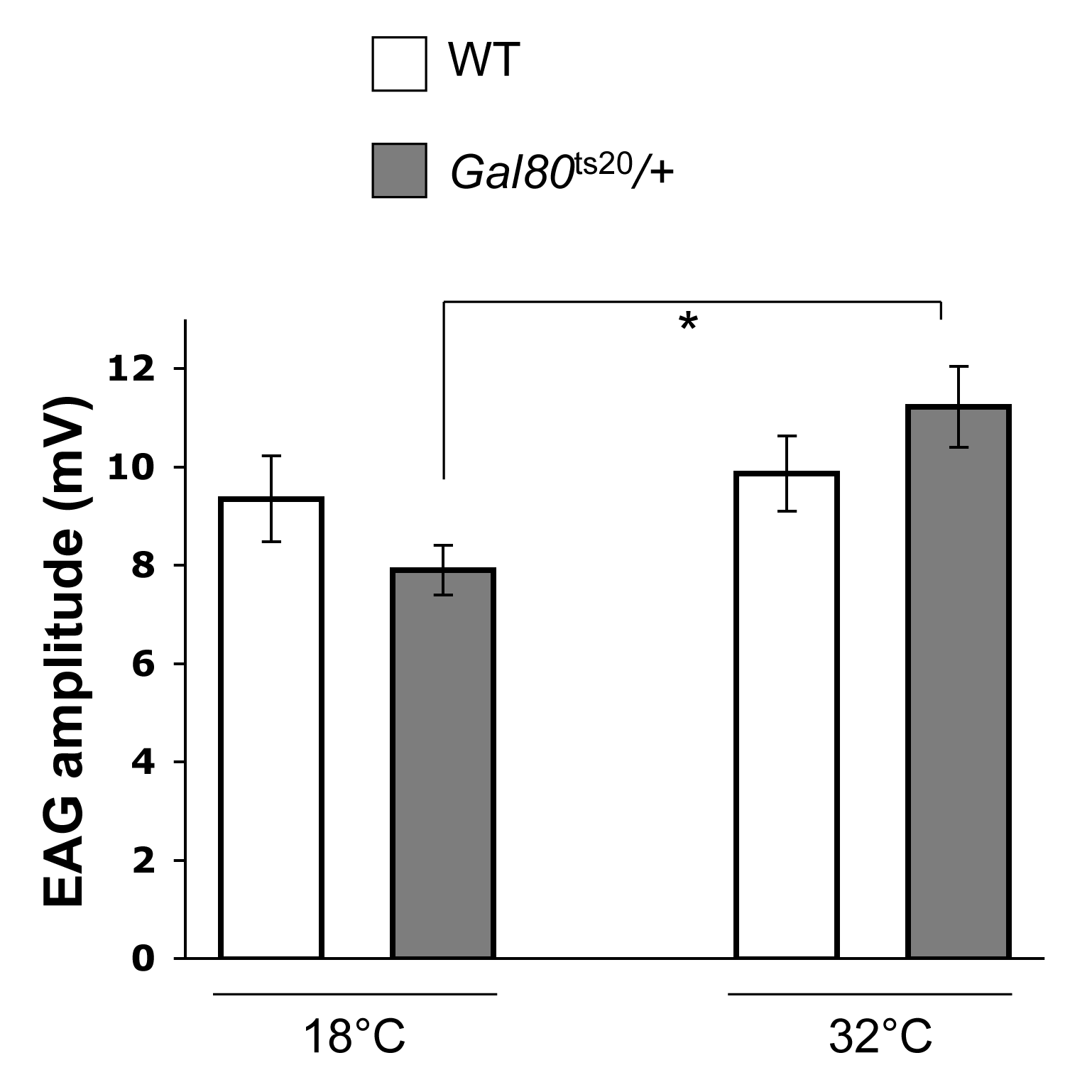

Supplement: Additional file 1 — Genetic background of Gal80ts20 transgene causes a temperature-dependent increase in EAG amplitude. EAG responses to ethyl acetate in wild-type controls are not different (p > 0.65) at 18°C and 32°C. EAG responses to ethyl acetate in Gal80ts20/+flies are significantly (p < 0.005) higher at 32°C than 18°C. For each genotype and temperature, at least 8 EAG recordings from minimum 4 flies were analyzed. Asterisks denote a significant (p < 0.05) change. All values are mean ± S.E.M. [file 1472-6793-9-22-S1.tiff]

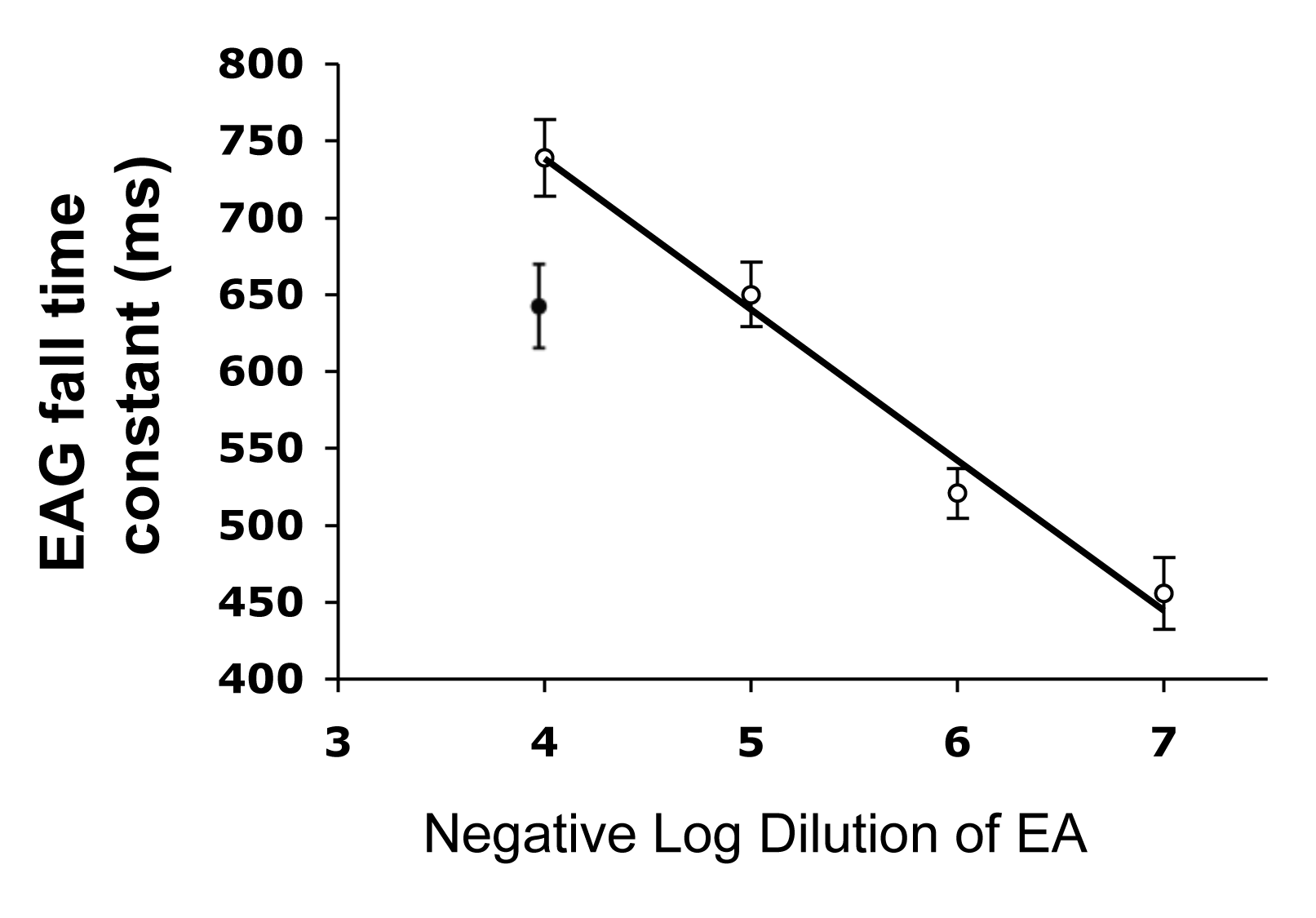

Supplement: Additional file 2 — EAG Fall Time Constant is a function of odor intensity. The decay of EAG response in Gal80ts20/Or83b-Gal4; UAS-PTX/+ flies at 18°C becomes faster as the concentration of ethyl acetate (EA) decreases (open circles). The EAG τf in response to a 10-4 dilution of ethyl acetate in Gal80ts20/Or83b-Gal4; UAS-PTX/+ flies at 32°C (closed circle) is equivalent to that of Gal80ts20/Or83b-Gal4; UAS-PTX/+ flies at 18°C in response to a 10-5 dilution of ethyl acetate. For every dilution of ethyl acetate, at least 8 EAG recordings from minimum 4 flies were analyzed. All values are mean ± S.E.M. [file 1472-6793-9-22-S2.tiff]

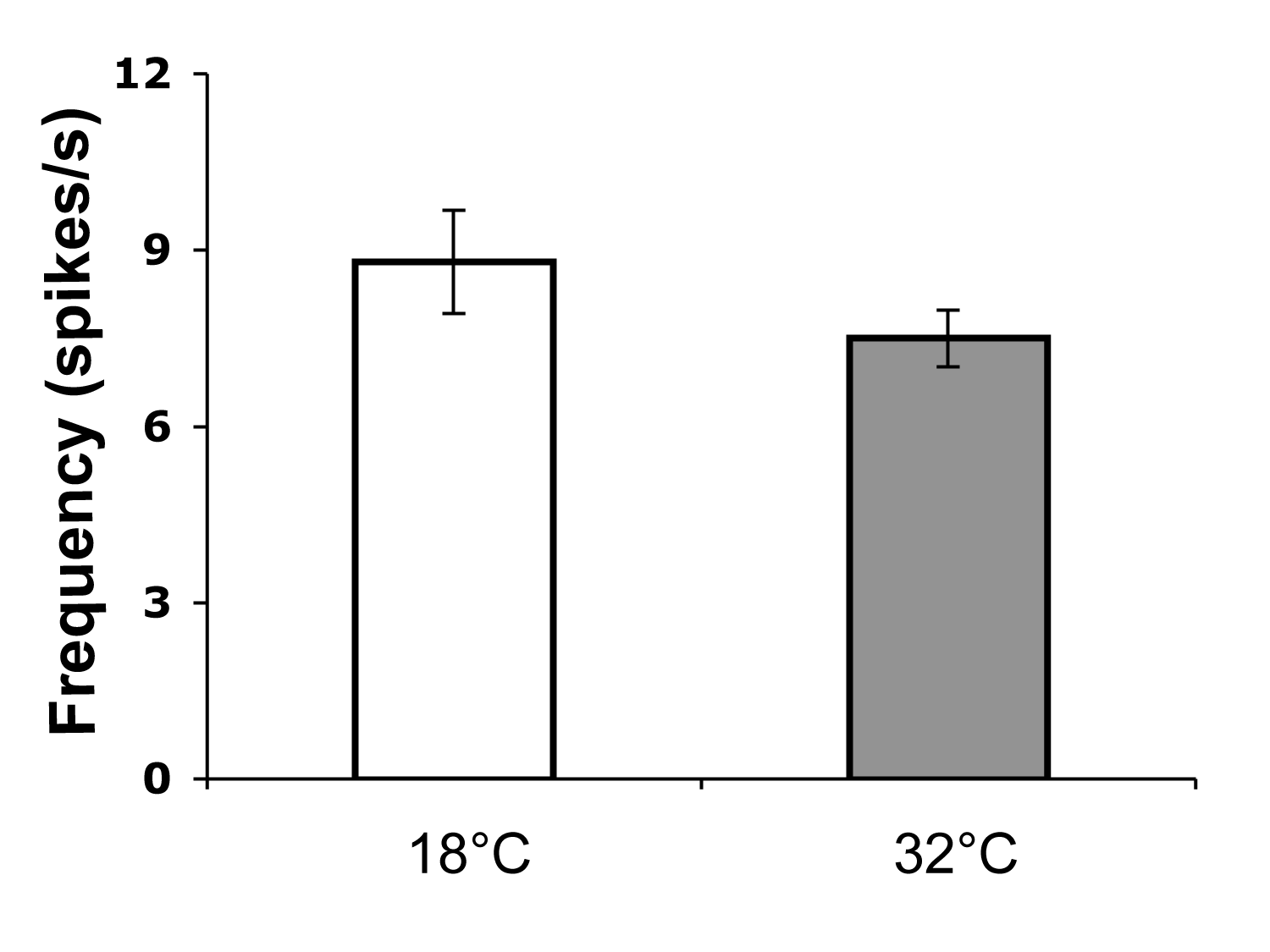

Supplement: Additional file 3 — Go inactivation does not alter spontaneous firing frequency. The frequency of spontaneous spikes in Gal80ts20/Or83b-Gal4; UAS-PTX/+ flies is not different (p > 0.21) when Go signaling is intact (18°C) or blocked by PTX expression (32°C). For each genotype and temperature, responses from at least 8 ORNs from minimum 4 flies were analyzed. All values are mean ± S.E.M. [file 1472-6793-9-22-S3.tiff]
